# Comparative Study of the Effects of Salinity on Growth, Gas Exchange, N Accumulation and Stable Isotope Signatures of Forage Oat (*Avena sativa* L.) Genotypes

**DOI:** 10.3390/plants9081025

**Published:** 2020-08-13

**Authors:** Syed Sadaqat Shah, Zhijian Li, Hong Yan, Lianxuan Shi, Bangwei Zhou

**Affiliations:** Key Laboratory of Vegetation Ecology, Ministry of Education, Institute of Grassland Science, Northeast Normal University, Changchun 130000, China; shuhangafridi@gmail.com (S.S.S.); yanh603@nenu.edu.cn (H.Y.); lianxuanshi@nenu.edu.cn (L.S.)

**Keywords:** forage type oat, salinity stress, gas exchange, stable isotopes, N accumulation

## Abstract

Identifying suitable salt stress-tolerant phenotypes based on their agronomic and physiological traits remains a herculean task in forage-type oat (*Avena sativa* L.) breeding. This study examined the responses of six forage-type oat cultivars under four levels of saline stress over the vegetative growth cycle. Crop growth, water status-related traits and nitrogen status-related traits were analyzed in different plant parts to evaluate effective approaches for identifying salt tolerance. Plant biomass, height, tiller number and culm thickness changed substantially during salinity, but they were not precise enough for use in estimating genotypic salinity tolerance during long-term stress. Genotypes bearing larger numbers of tillers showed greater sensitivity to salinity due to its effects on biomass loss. Tolerant genotypes exhibited higher relative shoot biomass together with higher water use efficiency. The concentrations of Na^+^, K^+^ and their ratio, combined with the δ^13^C in shoots and roots were effective indicators for estimating tolerant genotypes through better water maintenance. N concentrations of shoots were the most efficient for evaluating genotypic tolerance. Low nitrate reductase (NR) and glutamine synthetase (GS) activity might be key factors limiting N accumulation. Chlorophyll (Chl) content and net photosynthetic rate, as well as stomatal conductance and evaporation, were useful for identifying salinity tolerance physiological mechanisms, but the effectiveness was low for genotypic tolerance testing for forage type oats due to the interaction between genotypes and salinity levels. The selection of high salinity-tolerant genotypes should focus on genotypes with photosynthetic resilience to salt, followed by high N metabolism (higher NR and GS activities) to ensure accumulation of more N in the shoot dry matter.

## 1. Introduction

Oat (*Avena sativa* L.) is an important cereal and forage crop, making it a double-purpose crop, and is widely planted in low fertility or saline-alkaline soil. China is one of the largest forage crop consumers in the world, with the importation of oat hay increasing steadily from 0.15 million tons in 2008 to 0.39 million tons in 2017 from the United States, Australia etc., and oats are the second largest imported forage crop, followed by alfalfa [1]. By contrast, vast areas of low-fertility farmland, such as the Northeast China Plain and the Qinghai Tibet Plateau, with their high salinity and alkalinity (accounting for 6.62% of the total arable land), are restricted from large-scale cultivation of oats due to a lack of salt-tolerant varieties [2]. Breeding for salt-tolerant oats that could be widely planted across large areas of low fertility would not only increase the production of fodder grasses and ensure fodder supplies to local livestock industries, but would also increase soil cover in early spring in northern China, decreasing wind-borne dust and alleviating pressure on the local environment. An effective phenotyping approach remains a worthwhile exercise in breeding for salinity tolerance.

Although several studies have analyzed genotypic variation of oats under salinity for estimating morphological and physiological traits, the majority of them were focused on the grain yield. However, the genotypic response of forage type oats (usually harvested after anthesis) under salinity remains ambiguous. Under traditional methods of breeding, salt-tolerant germplasm selection remains very slow, with the reality that breeders need to identify salt-tolerant germplasm with “experienced eyes” [3]. Plants under saline conditions exhibit dehydration (i.e., water deficit), which is indicated by lower water status due to increased difficulty in taking up water from the soil, or a low capacity to maintain water inside the plant [4]. Salinity may cause a decrease in stomatal conductance (g_s_) and evaporation (*E*), reducing the effectiveness of carbon assimilation due to the amounts of toxic ions absorbed by the root surface and into the plant [5]. In the long term, salinity causes water stress and ion toxicity, which affect plant growth via reduced photosynthetic rates, carbon assimilation, tissue expansion and cell numbers, and prohibit nitrogen metabolism by reducing the content and activity of key metabolic enzymes (e.g., nitrate reductase, glutamine syntheses) [6,7]. The use of the stable isotope of carbon to evaluate genotypic abiotic stress tolerance has been widely applied in several crops (e.g., wheat, rice, barley) [5,8]. The stable isotopes can act as a time-integrated indicator of how plants interact with and respond to their environment, and indicate genotypic differences when evaluating crop stress tolerance [8]. For example, carbon isotope composition (δ^13^C) presented a clear advantage compared to gas exchange measurements during the evaluation of wheat water and nitrogen availability, because it provided information on the long-term plant photosynthetic and transpirative performance, which was less labor-intensive than gas exchange measurements [9,10]. However, this method has rarely been reported for evaluation of oat salinity tolerance, especially for forage type plants.

Under saline conditions, N metabolism is known to be restricted due to retardation of N uptake and reductions in NO_3_^−^ levels and NH_4_^+^ assimilation, leading to a severe decline in crop N accumulation [11]. In addition to disruption of water availability and absorption, inhibition of NO_3_^−^ uptake by Cl^−^, low NO_3_^−^ loading into the root xylem, alteration in the activities of N assimilating enzymes, decreases in transpiration and reductions in relative growth rate affect the N metabolism of salinity-stressed plants [12,13]. Nitrate reductase (NR; Enzyme Commission 1.6.6.1) and glutamine synthetase (GS; Enzyme Commission 6.3.1.2) are the two key enzymes required for N assimilation and associated with carbon metabolism [7]. Experiments conducted in many plant species (e.g., microalgae, sunflower, soybeans) have clearly associated the activities of NR and GS with plant growth rate [7,14,15]. GS is an enzyme that plays an essential role in the metabolism of nitrogen by catalyzing the condensation of glutamate and ammonia to form glutamine, during which GS activity is influenced by the ammonium ion concentration and water, depending on their binding affinities [16].

N isotope composition (δ^15^N) is linked to N metabolism in plants and is a useful tool for estimating abiotic stress tolerance and its associated genotypic differences (to salinity and drought); however, there is little literature available on the underlying biochemical mechanisms [17,18,19]. N fractionation occurs during translocation between organs and is also related to N metabolism during uptake, assimilation, recycling and redistribution of N within the tissue [6]. Both GS (deals with assimilation of ammonium) and NR (deals with nitrate assimilation) are the key enzymes that usually discriminate against ^15^N, especially when the N supply is lower than the N demand of a plant [20]. Even variation in δ^15^N has been considered as an indicator of the crop N status in response to salinity, with a substantial number of reports indicating that ^15^N fractionation is diverse, depending on the growing conditions and genotypic resilience [6,10]. Therefore, a comprehensive physiological understanding of how the C and N isotope signatures vary in response to the interaction between salinity stresses and genotypes is of paramount importance for breeding stress-tolerant cultivars.

Much research has been conducted on phenotyping cereal-type oats and examining genotypic tolerance to salinity, whereas there has been little work undertaken on forage type oat, especially during the pre-harvest stages. Moreover, information is scarce on the agronomic and physiological traits that combine the N and C isotopic signature responses under varying salinity in forage type oat. In our current experiments, six oat genotypes with different growth performance were grown at different salinity levels to test the stress effect on growth, gas exchange, ion accumulation, the activity of NR and GS, and the isotopic signatures of C and N. The selected genotypes have been cultivated locally. They exhibited similar plant phenology and different agronomic traits and growth performance under combined sandy and slightly saline field conditions [21].

## 2. Results

### 2.1. Biomass and Related Growth Traits

**Figure 1:**
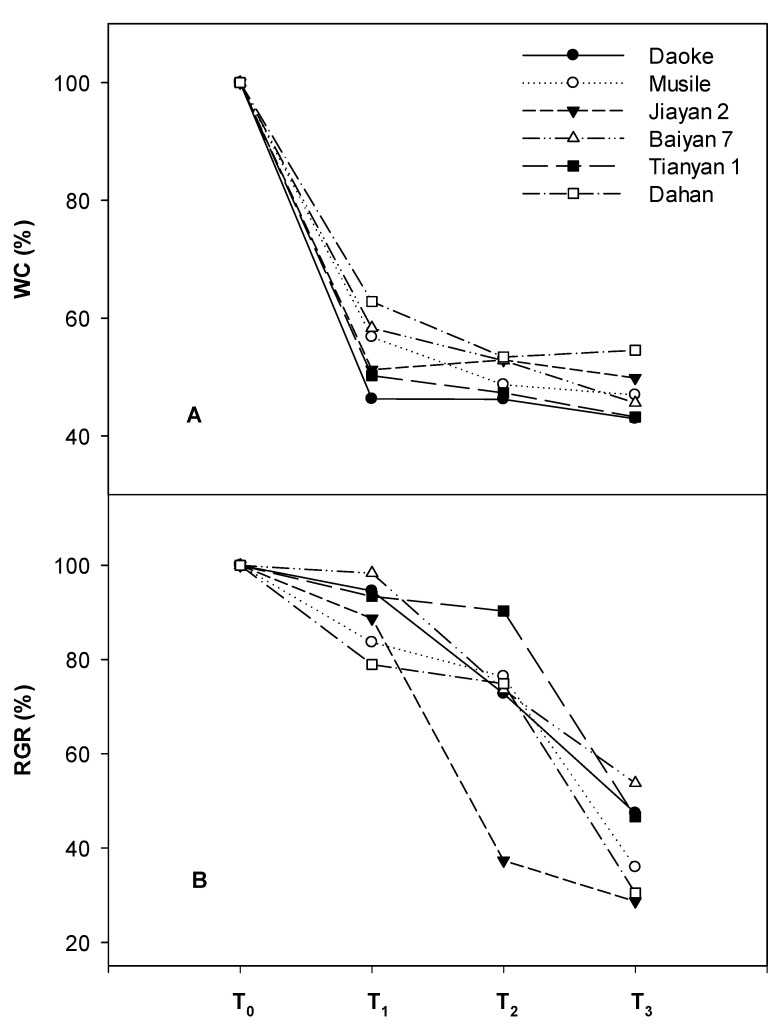
Relative decrease in water content (WC) (**A**) and growth rate (RGR) (**B**) of six different oat genotypes under different salinity concentrations compared to control conditions. T_0_ (Control, normal Hoagland solution); T_1_ (Hoagland solution with additional NaCl, EC at 8 dS m^−^^1^); T_2_ (Hoagland solution with additional NaCl, EC at 16 dS m^−1^); T_3_ (Hoagland solution with additional NaCl, EC at 24 dS m^−1^).

All the agronomic traits, including shoot biomass, plant height, tiller number and culm thickness were significantly and negatively affected by the different levels of salinity (Table 1). Plant biomass, height and tiller number interacted and changed significantly with salinity. The genotypic effect of biomass was significantly different under control conditions, which showed that the genotypes Jiayan 2 and Daoke yielded the highest biomass, while the relative biomass of these two genotypes decreased rapidly under salinity. The genotypes Dahan, Musile, Tianyan 1 and Baiyan 7 maintained significantly higher relative biomass under the mild stress treatment (Table 2). The growth of Tianyan 1 was fastest under moderate salinity due to its natural height contributing substantially to the formation of biomass, while Jiayan 2 exhibited the lowest relative biomass under mild stress, and the relative biomass of Daoke was also low, but not significantly different from the other genotypes. The relative growth rate (RGR) per day changed significantly among genotypes and salinity treatments, but there were no interactions between them. Jiayan 2 and Daoke, together with the short shoot genotype Dahan, had significantly lower RGRs relative to the other genotypes. The RGR was less affected by mild (12.5% reduction from control levels) and moderate (25% reduction from control levels) salt stress than the severe treatments (62.5% reduction from control) (Figure 1).

### 2.2. Gas Exchange Parameters and the Related Water Status

**Figure 2:**
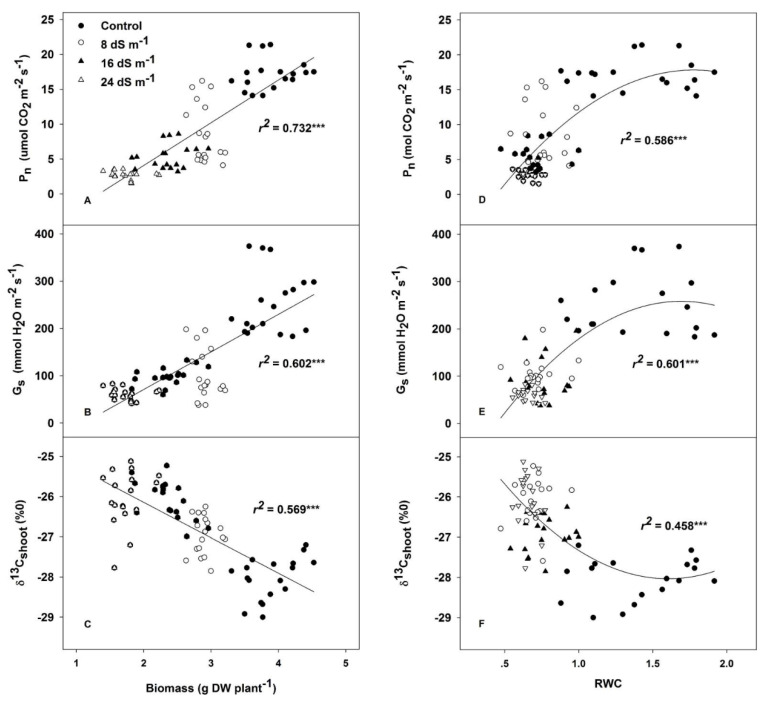
The relationship of shoot biomass to leaf net photosynthetic rate (P_n_) (**A**); stomatal conductance (g_s_) (**B**); and stable carbon isotopes (δ^13^C_shoot_) (**C**). The relationship of relative water content (RWC) to leaf net photosynthetic rate (P_n_) (**D**); stomatal conductance (g_s_) (**E**); and stable carbon isotopes (δ^13^C_shoot_) (**F**). Each point represents the individual value for a given replicate and genotype within each growing condition.

Although the trend in leaf relative water content (RWC) was not clear among genotypes, this parameter was tremendously sensitive to changes in salinity, even for mild stress, which was proven by the fact that 47% of the water had disappeared in the mild salinity treatment compared to the control (Table 3). The net photosynthetic rate (P_n_) of the expanded leaves, the stomatal conductance (g_s_) and the rate of transpiration (*E*) varied significantly among genotypes, and reduced as the salinity increased. However, the significant G × T interaction indicated that genotypic differences should be examined for each growing condition independently. Intrinsic water use efficiency (WUE_i_) changed significantly among all genotypes and salinity levels, exhibiting differences among treatments, but no interaction with different genotypes. Although the ratio of intercellular to ambient CO_2_ concentration (C_i_/C_a_) responded to the salinity treatments, there were no clear genotypic differences. The salinity treatments significantly increased the stable carbon isotope composition (δ^13^C) in the dry matter of shoots. Dry matter δ^13^C changed significantly among genotypes in both shoot and root samples. The δ^13^C of Tianyan 1 shoots was higher than in the other genotypes in all the treatments, while the change in the pattern of root δ^13^C was not clear among genotypes and salinity treatments. Across the treatments and genotypes, shoot biomass was strongly and positively correlated with P_n_, and g_s_ in leaves (*r*^2^ = 0.732, *p* < 0.001; *r*^2^ = 0.602, *p* < 0.001; respectively) (Figure 2A,B), and negatively associated with δ^13^C in shoots (*r*^2^ = 0.569, *p* < 0.001), but it was not affected by δ^13^C in roots (Figure 2C). Similarly, RWC was positively associated with P_n_ and g_s_, and negatively associated with δ^13^C in leaves in the salinity treatments, and exhibited a saturation response in control conditions (Figure 2D–F).

### 2.3. Nitrogen Concentration, Nitrogen Stable Isotopes and Related Enzymes

The interactions between salinity and genotypes were not significant for stable isotopes of nitrogen (δ^15^N) and N concentration in either shoots or roots (Table 4). The δ^15^N of the shoots was significantly different among treatments, showing its highest values in control conditions. Similarly, the δ^15^N of roots revealed significant differences among genotypes but no significant differences in response to salt stress treatments. The leaf Chl content was slightly affected by the mild and moderate salinity treatments and was greatly decreased in the severe stress treatment. Both NR and GS activities significantly decreased with the increase in salinity stress, but only NR showed significant genotypic differences among genotypes. The genotype Tianyan 1 presented the highest N concentration and the lowest NR activity with the highest biomass under mild and moderate stress conditions (Supplementary Table S1). GS activity decreased with increasing salinity, but it only showed genotypic effects in the control treatment (where it was significantly higher for genotypes Biayan7 and Dahan) and the mild salinity stress treatment (where it was significantly higher for genotypes Tianyan 1 and Musile) (Supplementary Table S1).

**Figure 3:**
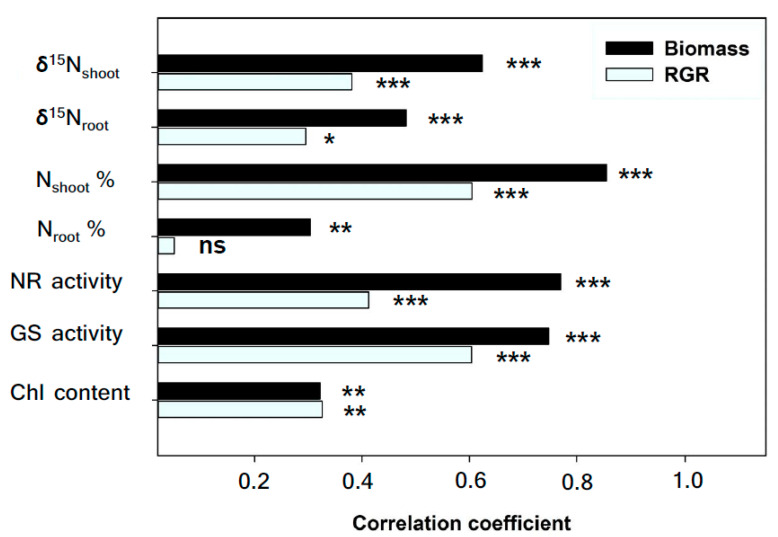
Correlation coefficients of the nitrogen stable isotope composition (δ^15^N) and N concentration of dry matter in shoots and roots, nitrate reductase activity (NR), glutamine synthesis activity (GS) and chlorophyll content (Chl) in leaves with total shoot biomass and relative growth rate (RGR) across treatments and genotypes. Levels of significant are as follows: ns, not significant; * *p* < 0.05; ** *p* < 0.01 and *** *p* < 0.001. *n* = 72.

**Figure 4:**
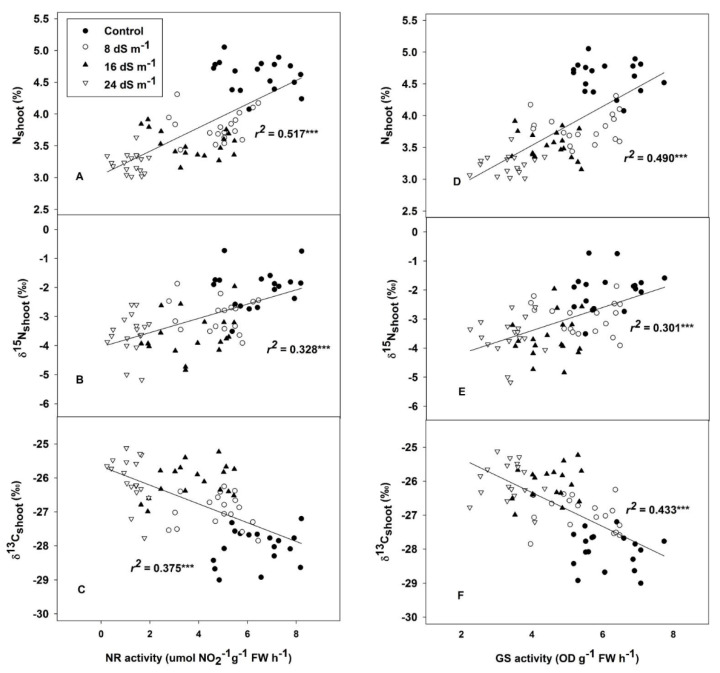
The relationship of nitrate reductase (NR) activity with nitrogen concentration (N%) (**A**); nitrogen isotope composition (δ^15^N) (**B**); and carbon isotope composition (δ^13^C) (**C**). The relationship of glutamine synthetase (GS) activity to nitrogen concentration (N%) (**D**); nitrogen isotope composition (δ^15^N) (**E**); and carbon isotope composition (δ^13^C) (**F**), in oat leaves across all treatments and genotypes. *** *p* < 0.001.

Relationships between biomass, RGR and traits related to N status were assessed independently across treatments and genotypes. Overall, the biomass was strongly positively correlated with δ^15^N in shoots and roots, N concentration and GS and NR activity in leaves, and also positively correlated with N concentration in roots and Chl in leaves (Figure 3). Meanwhile, the RGR was strongly and positively associated with δ^15^N in shoots, the N concentration and GS and NR in leaves, and also positively correlated with the δ^15^N in shoots and Chl content in leaves, but no relationship to N concentration was observed in roots (Figure 3). GS and NR activities were positively correlated with N concentration and δ^15^N in leaves (Figure 4A,B,D,E), but they were negatively associated with δ^13^C across treatments and genotypes (Figure 4C,F).

### 2.4. Ion Concentration Determination

**Figure 5:**
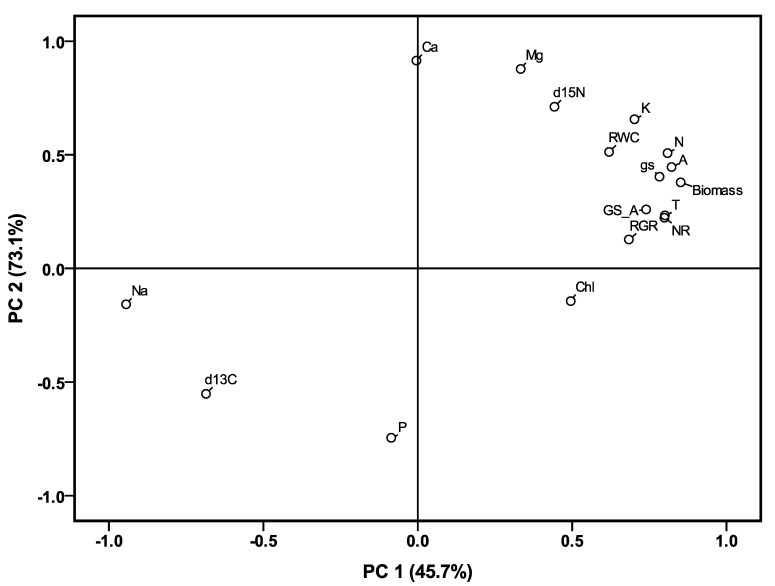
Principal component analysis (PCA) of biomass, RGR, RWC and the traits related to water, N and ions in the shoots in the salinity treatments. All abbreviations are as defined in Table 3, Table 4 and Table 5.

The effects of the salinity treatments on ion concentrations were significant for Ca^2+^, Mg^2+^, K^+^, Na^+^ and P in both shoots and roots (Table 5). Compared to control conditions, the treatments significantly increased the concentration of Na^+^ and decreased Mg^2+^, Ca^2+^ and K^+^ in all plant parts. The P concentration was increased in the shoots by the salinity treatments but without a clear trend, however, it did not change among the control or mild and moderate treatments in the roots. Moreover, the K^+^/Na^+^ and Ca^2+^/Na^+^ ratios in both shoots and roots also significantly decreased with increasing salinity (Table 5, Supplementary Table S2), but they were not altered significantly in the mild, moderate and severe saline treatments in either of the plant organs. A principle components analysis was performed to estimate the contribution of ion concentration, which explained the biomass, RGR and RWC across treatments and genotypes (Figure 5). The two first components explained 72.6% of the total variation. Biomass, RWC and RGR were located close to each other and were positively associated with Mg^2+^, K^+^, and N concentrations in shoots and Mg^2+^, K^+^ and Ca^2+^ concentrations in roots, and were negatively affected by the Na^+^ concentration in both shoots and roots.

### 2.5. Overall Effects of Treatments and Genotypes on Agronomic and Physiological Traits

In order to test the association between shoot biomass and physiological traits including water status, N status, the water and N traits combined and the total parameters, a multiple linear regression (stepwise) analysis was performed by using shoot biomass as the dependent variable (Table 6). In this case, all the physiological traits were selected as variables to evaluate the variability in shoot biomass. In the water status traits, the traits chosen by the model were P_n_ followed by E, δ^13^C_shoot_ and δ^13^C_root_, and collectively explained 82% of the variability in biomass. However, N_shoot_ was chosen as the first explanatory variable in the N status traits and combined with NR_activity_ and GS_activity_ explained 79% of the variability in shoot biomass. The final stepwise analysis consisted of traits associated with water or N traits together, and we assessed the contribution of both group traits to evaluate the variability in the biomass. Nevertheless, with both water and N traits combined, the first variable chosen was N_shoot_, and the second was P_n_ followed by GS_activity_ and δ^13^C_root_, with all of these traits explaining 83% of the variability in shoot biomass. Moreover, when the ion concentrations in plants were combined, the Na^+^ concentration in shoots was the first trait that dominated the variation in biomass, the second was Mg^2+^ in shoots followed by Na^+^ concentration in roots, δ^13^C_root_ and P_shoot_ were chosen as the fourth and fifth variables by the model, and all of these variable collectively explained 90% of the variability in shoot biomass.

**Figure 6:**
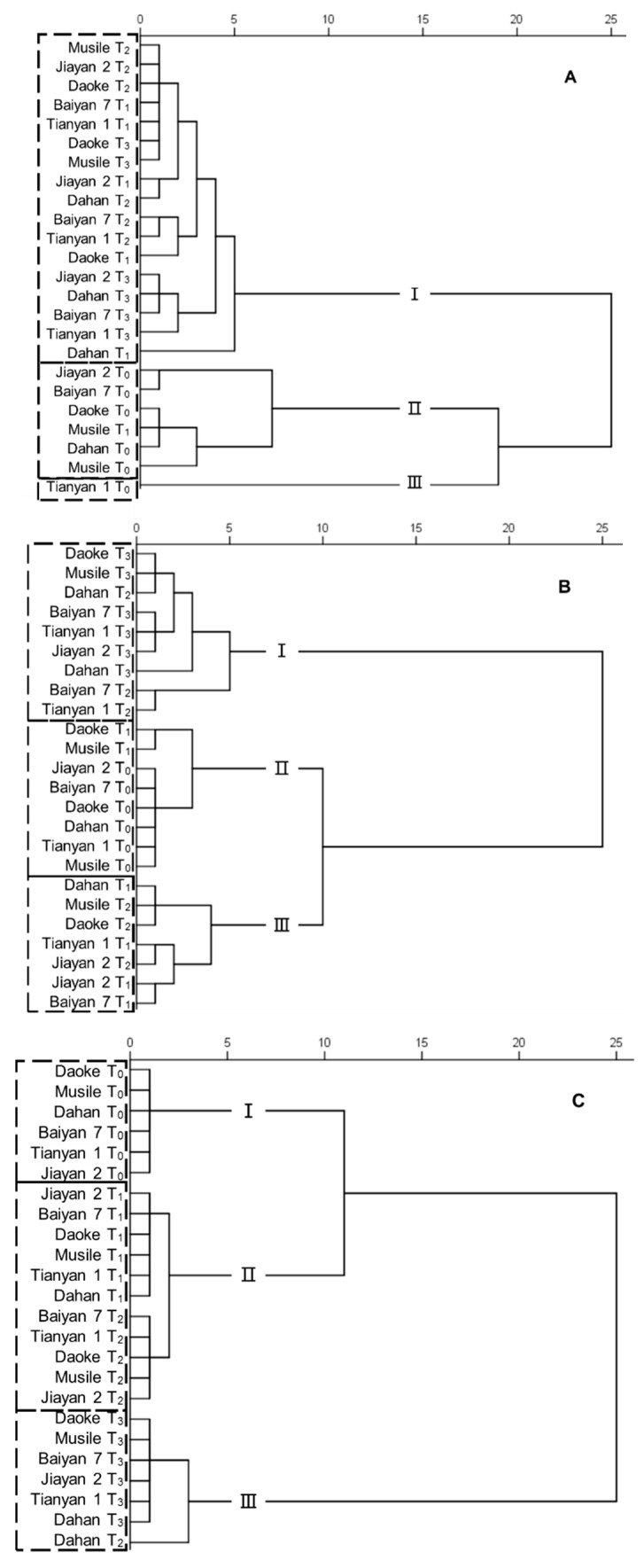
Cluster analysis of six oat genotypes grown in the given treatments. Variables were chosen from physiological traits studied in this work, including the water status traits P_n_, g_s_, *E*, WUE_i_, VPD, C_i_/C_a_ and δ^13^C (abbreviations defined in Table 2) (**A**); the N metabolism traits δ^15^N, N concentration, NR and GS activity (abbreviations defined in Table 3) (**B**); and Ca^2+^, Mg^2+^, K^+^, Na^+^, and P concentrations (**C**).

In order to categorize the genotypes on the basis of the evaluated traits, cluster analyses were conducted on traits relating to gas exchange, N metabolism and ion concentrations (Figure 6). In the cluster analysis for water status, the genotypes were sorted into three groups, with the control and salinity treatments well separated. Tianyan 1 was sorted into a single group, as this genotype possessed a higher water status than the other genotypes in control conditions. The genotype Musile under mild saline conditions had lower biomass and higher P_n_, g_s_ and E, but sorted in the same water status as the control plants (Figure 6A). In the cluster analysis for N metabolism, the genotypes were sorted into three groups. The genotypes Daoke and Musile under the mild and moderate saline treatments were placed with their corresponding lower stress level groups, while the genotypes Dahan, Baiyan 1 and Tianyan 1 under moderate salinity stress were classified into the most severe stress group (Figure 6B). The cluster analysis based on ion concentrations displayed an almost perfect separation among treatments, although genotypes in the mild and moderate treatments were classified in the same group. The genotype Dahan accumulated the lowest K^+^/Na^+^ ratio under moderate stress, but sorted closer to the high salinity stress group (Figure 6C).

## 3. Discussion

Assessment of appropriate agronomic traits is critical for current and future breeding efforts to improve oat grain yield under soil salinity and drought [6]. However, one of the primary purposes in breeding forage oats has been to select the genotype with an enhanced biomass based on its capacity to tolerate salinity. The tested oats growing under salinity stresses were acclimatized to a set of morphological, physiological and biochemical changes. Based on the strong interaction between the stress treatments and genotypes, distinguishing the genotypic salt tolerance capacity according to the growth and agronomic traits is quite challenging due to a set of unconfirmed variables. Salinity inhibited the growth of oat biomass and limited the plant height, tiller number and culm thickness. The same effects have been noted in many C_3_ species exposed to salinity (e.g., wheat, barley, brome) [22,23,24]. In optimal conditions, high biomass genotypes (for example Daoke and Jiayan 2) produced more tillers and greater plant height, but these two genotypes showed severe biomass loss under salinity due to reductions in tiller numbers and slower growth. The genotypes with moderate height and fewer tillers (for example Baiyan 7, Tianyan 1 and Musile) were not greatly affected due to greater resilience of the main stem than the later emerging secondary tillers [25]. According to the results, the shoot biomass was affected by different agronomic components under different salinity conditions. The biomass was mainly accumulated as increased culm thickness under control conditions, but it was affected by a decline in tiller number in mild salinity, and stagnating plant height under moderate salinity. Under severe salinity the factors affecting biomass could not be distinguished among the agronomic traits. Shoot biomass, relative growth rate and relative water content were strong indicators for determining the salinity tolerance of various genotypes.

Physiological traits exhibiting genetic variability under salinity stress have been widely reported [7,23]. Decreases in gas exchange traits (P_n_, g_s_ and *E*) following increases in salinity stress have been commonly observed in many crops grown under pot conditions (e.g., oat, barley, durum wheat) [26,27]. However, even though gas exchange parameters in oats changed significantly for both genotype effects and salinity stress, the strong interaction between them indicated that these parameters could not differentiate between tolerant and susceptible genotypes under varying salinity conditions. The effectiveness of P_n_ evaluated for biomass was higher than g_s_ and *E* under long-term salinity, which might be because oats exposed to heavy saline conditions are affected predominantly by diffusion and biochemical capacity (e.g., changes in the electron transport system, inhibition of Calvin cycle enzymes), and stagnating CO_2_ assimilation [7,28], rather than water limitations. Previous studies have also suggested that high g_s_ may be the most effective method of identifying genotypic tolerance in saline soil [6]. This may be due to the fact that tolerant genotypes have higher numbers of open stomata and may use Na^+^ instead of K^+^ for stomatal movements, or might reduce stomatal density to conserve water when grown under saline conditions [29]. Under long-term salinity, the genotypes with relatively higher biomass clearly possessed higher WUE_i_ than the other genotypes under moderate stress. In other words, salinity tolerance is mainly the consequence of high WUE_i_.

In many C_3_ species, δ^13^C has been considered as a key trait that balances interaction over time in many biochemical processes (e.g., opening and closing of stomata to regulate C_i_/C_a_, fractionation of C_13_ and C_12_ by key photosynthetic enzymes), and has been used to estimate genotypic drought and salinity tolerance under drought conditions for wheat, barley, sugar beet etc. [30,31,32]. In the current work, the increase in shoot δ^13^C of higher biomass exhibited low g_s_ activation, which implies that increased long-term g_s_ limitation under salinity caused higher δ^13^C. The increases in the δ^13^C of salt stressed leaves relative to the control might have been associated with a low C_i_/C_a_ ratio, which in turn was caused by stomatal closure and a slowdown in CO_2_ diffusion and biochemical assimilation of CO_2_ in the mesophyll cells inside the leaves [33], and may also have been associated with key carbon fixation enzyme activities (e.g., Rubisco). The lower biomass genotypes (Musile, Tianyan 1 and Baiyan 7) exhibited lower shoot δ^13^C in the control, and had greater ^13^C stomatal discrimination relative to the two high biomass genotypes (Daoke and Jiayan 2), thus hinting that the three former varieties were more tolerant than the other two. Although the correlation coefficient of δ^13^C with biomass was lower than for P_n_ or g_s_, the interaction of genotypes and salinity treatments was not significant in either shoots or roots, which confirmed that δ^13^C was a better indicator for evaluating genotypic tolerance to variable salinity. The salt-tolerant genotypes differed from the salt-sensitive genotypes not only due to a higher shoot biomass, but also a better water status, especially under moderate salinity conditions. The tolerant genotypes also exhibited a lower δ^13^C and higher P_n_, g_s_ and *E*. In previous studies, the δ^13^C in leaves has been positively correlated with biomass under a moderate salinity treatment (12 dS m^−1^) in durum wheat grown in pots [23], while a similar relationship was observed in saline conditions in the field in wheat cultivars [6]. Therefore, our results further highlight the advantage of δ^13^C as time-integrative trait over instantaneous gas exchange traits when evaluating genotypic salt tolerance.

In some C_3_ crops, Chl content is considered a biochemical marker of salt tolerance for genotypic identification, and relies on salt-tolerant genotypes showing increased or unchanged Chl content, while decreases in this parameter occur in salt-sensitive genotypes [34]. Under long-term salt stress, the degradation of Chl content per unit leaf area is caused by disruption of Chl synthesis and acceleration in its breakdown, which may be due to loss of photoprotection mechanisms [35]. In this work, apart from the high salinity treatment, there was no significant difference between the Chl content under saline and control conditions. This is quite different from other crops (e.g., wheat, barley) where leaf Chl content changes substantially due to salt sensitivity [23]. Indeed, the interaction of genotype and salinity stress was highly significant, which emphasized that it was infeasible to use Chl content to evaluate salt tolerance in oats.

Besides a direct osmotic effect on plant water availability, the parallels between the changes in biomass and N concentration suggested that salinity also stunted growth via inhibition of N metabolism. Salt interrupts plant growth through declining N availability and/or N metabolism, including losses in N uptake, chemical reduction of NO^3−^, and NH^4+^ assimilation [36]. In this experiment, NR activity, which is the key limiting factor during nitrate reduction, decreased under long-term salinity stress, and this corresponds to many reports in the literature where NR activity increased under short-term salinity but decreased thereafter [37,38]. Similar to NR, GS activity also decreased under salinity. Generally, the tolerant genotypes exhibited higher activities. This result is concomitant with previous studies on where decreases in NR and GS activity have been observed in response to salinity [7]. Indeed, inhibition of nitrate uptake rates exceeding 50% were observed under 60 mM NaCl in wheat [39], whereas ammonium uptake seemed much less affected [40]. A similar trend was also detected in the current work, but NR activity reduced by 41% under moderate (16 dSm^−1^) salinity conditions. This proves that oats are more saline-tolerant than wheat. Our results exhibited a strong interaction between genotypes and salinity treatments for NR and GS, and highlighted the fact that NR and GS activities are not reliable indicators for estimating genotype salinity tolerance. By contrast, the N concentration in shoot dry mater changed substantially among stresses and genotypes, and the tolerant genotypes Musile and Tianyan 1 exhibited significantly higher N concentrations in shoots (the same trend was observed in Daoke, but it was not significant) than in the other genotypes, which proves that tolerant oat varieties accumulated higher N concentration than the susceptible ones, resulting in faster growth [41].

A detailed study of the literature shows that the δ^15^N has been considered a key criterion for differentiating between wheat genotypes that are tolerant and susceptible to salinity and drought [23]. Compared with the control, the salinity treatment decreased the δ^15^N in both oat shoots and roots, indicating that N isotope fractionation was affected by salinity stress. During this process, NR and GS were the two enzymes that dominated δ^15^N fractionation and thus N accumulation. Low ^15^N/^14^N fractionation may occur during N uptake by roots and in many of the accumulation-associated metabolic processes; for example, lower NR and GS activity associate to a decrease in ^15^N fractionation [7]. The δ^15^N in shoots was higher than in roots for all treatments, which might have been due to stomatal closure caused by the low availability of water (supported by lower RWC, g_s_ etc.). Saline conditions would prohibit the loss of ammonia and nitrous oxide during translocation, exudation or volatilization, hence decreasing the δ^15^N [42,43]. Meanwhile, the potential high ^15^N fractionation may have occurred due to the low consumption of N under salt stress and the constant supplementation of external N in the substrate [44]. The positive correlations of δ^15^N with biomass and RGR might be due to the differences in δ^15^N across treatments being related to differences in N assimilation capacity and N demand. The same positive relationships have been noted in other work on wheat [45,46]. In fact, NR and GS activities and Chl content were associated with N status in the current study. The stable nitrogen isotope signature (δ^15^N) was an effective indicator of salinity stress in mild and moderate saline conditions.

Overall, this study highlighted the potential for ion concentrations (Na^+^, K^+^, Mg^2+^ or the ratio of them) to be used alone or together with water- and N status-related traits in shoots and roots to assess genotypic performance under salinity. The main parameters of biomass formation were determined by the Na^+^/Mg^2+^ balance in shoots, which plays a key role in genotypic performance under salinity. Indeed, when selecting for genotypes of higher biomass, a higher genotypic P_n_ in leaves along with lower δ^13^C in shoots and higher δ^13^C in roots represent sensitive models for identification of genotypes with better water status for tolerance of moderate salinity. This study also guides us in selecting a higher N concentration in leaves combined with higher NR and GS activity for genotypes tolerant for high salinity stress.

## 4. Materials and Methods

### 4.1. Plant Material and Growth Conditions

Six oat (*Avena sativa* L.) varieties (Dahan, Musile, Tianyan 1, Baiyan 7, Daoke and Jiayan 2) were selected that have been popularly cultivated as forage crops in recent years across northern China. These genotypes were selected on the basis of information available about their genetic diversity and salinity tolerance in the field [47]. The experiment was conducted in a greenhouse under sunlight at the Northeast Normal University starting from the 5 May 2018. The seeds were soaked with distilled water in petri dishes at 4 °C in a controlled temperature chamber for vernalization. One week later the seeds were planted in 30 cm × 30 cm × 30 cm plastic containers (with holes at the bottom) filled with well-washed sandy clay combined with vermiculite. A completely randomized design was used in this experiment (six genotypes × three replicates per genotype × four salinity treatments) (*n* = 72). Five plants were maintained in each container after emergence. Half-strength Hoagland solution was provided for 1 week after germination [48], and was subsequently increased to full strength until harvest. The same amount of Hoagland solution was provided to each container following each full irrigation. After a month of growth, when the plants had reached the tillering stage, four different salinity treatments were imposed: (1) T_0_ (control, irrigated with normal Hoagland solution, EC at 1.8 dS m^−^^1^); (2) T_1_ (mild NaCl saline conditions, irrigated with saline Hoagland solution with EC at 8 dS m^−^^1^); (3) T_2_ (moderate saline conditions, irrigated with saline Hoagland solution with EC at 16 dS m^−1^); (4) T_3_ (high saline conditions, irrigated with saline Hoagland solution with EC at 24 dS m^−1^). The salinity concentration was increased progressively over 10 days by adding NaCl to the nutrient solution, starting from EC at 4 dS m^−1^ to reach the final salt levels. To avoid salt accumulation, the substrate was washed with abundant ground water every two weeks to remove the salinity. Overall, the final saline treatments were imposed for 40 days until the plants reached the heading stage when the high salinity conditions were too severe for growth, and then the total shoot biomass and root biomass was harvested.

### 4.2. Growth Parameters

Initially, single plants were harvested from each pot before NaCl treatments were conducted, and the fresh plant weight (*W*_1_) was assessed immediately using a balance. The samples were oven-dried until constant weight. After salinity treatment, another plant was harvested from each pot and the same weight evaluations were conducted as above. The equation used to calculate the relative growth rate (*RGR*) was [49]:

(1) $$RGR=\frac{In\;W_{2}-In\;W_{1}}{t_{2}-t_{1}}$$

where, *In*: natural logarithm; *W*_1_: dry/fresh weight of the plant at time one (in grams); *W*_2_: dry/fresh weight of the plant at time two (in grams); *t*_1_: the day before the salinity treatment was implemented; *t*_2_: the day the samples were harvested during the salinity treatment; *t*_2_ − *t*_1_ is the time interval in days between the harvests. In this paper, the *RGR* is expressed both in per day units (*RGR* per day) and in per control units (*RGR* per control).

Before all of the plants were harvested, three fresh leaf samples from each container were taken and the fresh weight was assessed immediately. Afterwards, the leaves were placed into distilled water in a beaker at room temperature for 4 h to reach full hydration. Turgid weight was immediately recorded after blotting. Finally, leaf samples were oven-dried for 48 h at 60 °C to calculate the dry weight. The equation to calculate the relative water content (*RWC*) is given below:

(2) $$RWC=\frac{FW-DW}{TW-DW}$$

where, *FW*: sample fresh weight; *TW*: sample turgid weight; *DW*: sample dry weight.

Plant height was measured with a ruler from the substrate to the top of the spike prior to harvest. Tiller number was recorded and culm thickness was measured using a Vernier caliper at the second internode from the surface of the substrate with three replicates in each container. A portable chlorophyll meter (Minolta SPAD 502 Meter, Plainfield, IL, USA) was used to measure the chlorophyll content in the middle of the flag leaf blade prior to its use in photosynthetic gas exchange. The shoots and roots were harvested from each container and oven-dried until constant weight. The shoot and root biomass are expressed as the mean weight of each plant.

### 4.3. Photosynthetic Gas Exchange Measurements

Before the plants were harvested, photosynthetic gas exchange measurements were conducted in three upper leaf blades from each pot using a LICOR 6400 leaf chamber connected to a portable infrared gas analyzer. Determinations were conducted at 400 µmol mol^−1^ CO_2_, 25 °C, and 50% relative humidity (RH) while maintaining a light level similar to the growing conditions (approximately 1800 µmol·m^−2^·s^−1^ PPFD). The leaf net photosynthetic rate (P_n_), stomatal conductance (g_s_), transpiration (*E*), the ratio of intercellular to ambient CO_2_ concentration (C_i_/C_a_) and vapor pressure deficit (VPD) were recorded when the readings stabilized. Intrinsic water-use efficiency was defined as the ratio of instantaneous rates of P_n_ and *E* by relatively simple equations [50].

### 4.4. N Concentration and Stable Isotope Signatures in Total Organic Matter

After harvesting, whole aboveground shoot and root samples were oven-dried separately and ground to a fine powder. Stable isotope ratios of carbon (^13^C:^12^C) and nitrogen (^15^N:^14^N) as well as the nitrogen (N) concentration in dry matter were measured at the facilities of the Northeast Normal University, Changchun, China, by using an elemental analyzer (Thermo-Finnigan, Bremen, Germany) coupled with an isotope ratio mass spectrometer (Delta C IRMS, Thermo-Finnigan, Bremen, Germany) (EA-IRMS) in continuous flow mode. An approximately 2 mg quantity of finely powdered plant material was weighed, packed into tin capsules and loaded into an automatic sampler before EA-IRMS analysis. Stable isotope values were denoted in δ notation [51]:

(3) δC13=(C13/C12)sample(C13/C12)standard−1

where “sample” refers to plant material and “standard” to the Pee Dee Belemnite (PDB) calcium carbonate. International isotope secondary standards of known ^13^C/^12^C ratios (IAEA CH7 polyethylene foil, IAEA CH6 sucrose and USGS 40 L-glutamic acid) were used for calibration to a precision of 0.1‰.

For the δ^15^N of plant dry matter, the same formula as δ^13^C was used except for standards. In the nitrogen isotope formula, the standard referred to N_2_ in Air. Atropine was used as a system check in the elemental analyses of nitrogen. Isotope secondary standards of known ^15^N/^14^N ratios (IAEAN1 and IAEA N_2_ ammonium sulfate and IAEA NO_3_ potassium nitrate) were used for calibration of δ^15^N to a precision of 0.2‰.

### 4.5. NR and GS Enzyme Activity Determinations

In order to determine the NR enzyme and GS semisynthetic activity, a suitable amount of fully expanded upper leaves was washed with distilled water and dried with filter paper. After that they were kept at −20 °C for 30 min. Aliquots of frozen leaves (0.1 g) of each sample were ground in 1.0 mL extract at 4 °C. The extract was clarified by centrifugation at 4000 g for 10 min at 4 °C. The subsequent steps were carried out according to the instructions of a nitrate reductase (NR) activity assay kit (Solarbio, Beijing, China). Enzyme activity was determined by measuring the absorbance at 540 nm with a Cecil CE 7200 spectrophotometer (Cecil Instruments, Cambridge, UK). GS activity was measured according to the instructions of a glutamine synthetase (GS) activity assay kit (Solarbio). The OD values of GS were determined at 540 nm by using a Cecil CE 7200 spectrophotometer (Malmesbury, Wiltshire, UK).

### 4.6. Ion Concentration Analysis

A small sample (0.1~0.2 g) of the plant powder was digested with 3 mL of concentrated HNO_3_ at 120 °C for 5 min, 150 °C for 5 min and 180 °C for 20 min. After digestion, each sample was brought up to a 50 mL final volume with deionized water. To analyze the quantity of ions (Ca^2+^, Na^+^, K^+^, P and Mg^2+^) in shoots and roots, inductively coupled plasma-optical emission spectrometry (ICP-OES) (L3200RL, Perkin Elmer, Germany) was carried out as described elsewhere [23], at the School of Chemical Science, Northeast Normal University.

### 4.7. Statistical Analysis

The agronomic and physiological trait data were subjected to factorial analysis of variance (ANOVAs) to assess the effects of salinity on different oat genotypes and their interactions. A Tukey’s b test (*p* < 0.05) test was performed to compare means of different genotypes and treatments. Pearson correlation coefficients between different agronomic and physiological traits were measured using a bivariate correlation method. The agronomic and physiological traits were divided into four categories. Agronomic traits included biomass, plant height, tiller number and culm thickness, while the physiological traits were divided into three categories comprising photosynthetic traits (including δ^13^C, carbon concentration and total carbon concentration), N metabolism traits (including δ^15^N, nitrogen concentration and total nitrogen concentration), and ion concentrations. To distinguish the genotypic category and treatment groups of each trait category, the genotype–treatment combinations (i.e., six genotype crosses with four treatments) were tested with an unweighted pair group method using arithmetic mean (UPGMA) cluster analysis. Linear stepwise models were constructed across the genotypes to analyze the association between biomass and different physiological traits. The models were independent for each treatment to include or exclude the variables from the model, with *p* = 0.05. Figures were created by using Sigma-Plot 12.5 (Systat Software, Inc. Sigma-Plot for Windows). Data were analyzed using IBM SPSS Statistics 23.0.

## 5. Conclusions

In conclusion, salinity stress restricted the formation of shoot biomass due to the development of short plants, low tiller numbers and thin culm thickness. RGR and RWC were informative for separating the salinity tolerance according to genotype. Among the physiological traits, even though gas exchange traits were sensitive to salinity, they were less effective at estimating genotypic salinity tolerance due to the genotype and treatment interaction. By contrast, the δ^13^C of shoots and roots was an effective indicator of salinity levels and genotypic tolerance during long-term growth. N concentration was effective for assessing genotypes and salinity conditions, but it was limited by the genotypic NR and GS activities and their effects on N accumulation. Chl content had low effectiveness for genotypic tolerance identification since there were no differences for mild and moderate salinity levels to control. In fact, the selection of high salinity-tolerant genotypes should focus on genotypes with high photosynthetic activity or high water maintenance following stomatal closure, and high N metabolism to accumulate more N in the dry matter in the shoots.

## Figures and Tables

**Table 1:** Effect of various levels of salinity on plant biomass, plant height, tiller number per plant and culm measurements, while treatment values are the means of the 18 measurements. Means followed by different letters are significantly different (*p* < 0.05) according to Tukey’s-b test.

	Plant Biomass (g DW Plant^−1^)	Plant Height (cm)	Tiller Number (Per Plant)	Culm Thickness (mm)	RGR (Per Day)
**Genotypes (G)**					
Dahan	2.54 ^a^	60.11 ^abc^	2.83 ^a^	0.47 ^a^	0.04 ^ab^
Musile	2.55 ^a^	56.98 ^a^	3.17 ^a^	0.54 ^b^	0.07 ^c^
Tianyan 1	2.79 ^b^	65.89 ^d^	2.83 ^a^	0.52 ^b^	0.09 ^d^
Baiyan 7	2.85 ^b^	61.81 ^cd^	2.67 ^a^	0.52 ^b^	0.07 ^c^
Daoke	2.81 ^b^	58.30 ^bc^	3.25 ^a^	0.55 ^b^	0.06 ^bc^
Jiayan 2	2.80 ^b^	58.30 ^ab^	3.00 ^a^	0.53 ^b^	0.04 ^a^
**Treatments (T)**					
T_0_ (Control)	3.89 ^d^	71.35 ^d^	3.61 ^c^	0.58 ^c^	0.08 ^c^
T_1_ (8 dS m^−1^)	2.90 ^c^	64.96 ^c^	3.17 ^bc^	0.53 ^b^	0.07 ^c^
T_2_ (16 dS m^−1^)	2.36 ^b^	57.90 ^b^	2.94 ^b^	0.50 ^a^	0.06 ^b^
T_3_ (24 dS m^−1^)	1.73 ^a^	49.66 ^a^	2.11 ^a^	0.48 ^a^	0.03 ^a^
**Level of Significance**					
G	0.000	0.000	0.152	0.000	0.000
T	0.000	0.000	0.000	0.000	0.000
G × T	0.000	0.000	0.048	0.075	0.928

**Table 2:** Comparison of shoot biomass of six oat genotypes under control conditions and biomass relative to control. Values shown for control conditions (g) and different levels of salinity (%) are the means of three replicates. Means followed by different letters are significantly different (*p* < 0.05) according to Tukey’s-b test. The associated sum of squares and probabilities (ns, not significant; * *p* < 0.05 and *** *p* < 0.001) are shown.

Genotypes	Biomass (g)	Biomass Relative to Control (%)		
	**Control**	**T_1_**	**T_2_**	**T_3_**
Dahan	3.46 ^a^	82.24 ^b^	62.12 ^ab^	49.78 ^a^
Musile	3.63 ^ab^	76.52 ^b^	62.72 ^ab^	42.54 ^a^
Tianyan 1	3.73 ^ab^	79.64 ^b^	74.79 ^b^	44.10 ^a^
Baiyan 7	3.92 ^cd^	78.66 ^b^	61.20 ^ab^	40.20 ^a^
Daoke	4.22 ^cd^	67.86 ^a^	58.50 ^ab^	50.94 ^a^
Jiayan 2	4.38 ^d^	66.26 ^a^	48.23 ^a^	43.70 ^a^
Sum of square (G)	1.80 ***	650.13 ***	1089.22 *	268.4 ns

**Table 3:** Effect of various levels of salinity on the relative water content (RWC), leaf net photosynthetic rate (P_n_), stomatal conductance (g_s_), transpiration rate (*E*), intrinsic water use efficiency (P_n_/E, WUE_i_), the ratio of intercellular to ambient CO_2_ concentration (C_i_/C_a_) and the stable carbon isotope composition (‰) of shoots (δ^13^C_shoot_) and roots (δ^13^C_root_) of six oat genotypes. Genotype values are the means of 12 measurements, while treatment values are the means of the 18 measurements. Means followed by different letters are significantly different (*p* < 0.05) according to Tukey’s-b test.

	RWC (#)	P_n_ (μmol CO_2_ m^−2^ s^−1^)	G_s_ (mmol H_2_O m^−2^ s^−1^)	*E* (mmol H_2_O m^−2^ s^−1^)	WUE_i_ (μmol CO_2_ mmol H_2_O^−1^)	C_i_/C_a_ (µmol mol^−1^)	δ^13^C_shoot_ (‰)	δ^13^C_root_ (‰)
**Genotypes(G)**								
Dahan	0.81 ^a^	7.67 ^a^	90.50 ^a^	2.43 ^a^	3.19 ^b^	0.57 ^a^	−26.72 ^b^	−26.76 ^bc d^
Musile	0.88 ^a^	8.52 ^bc^	144.08 ^c^	3.67 ^b^	2.27 ^a^	0.61 ^a^	−26.84 ^b^	−27.15 ^a^
Tianyan 1	0.90 ^a^	8.78 ^c^	158.67 ^d^	3.63 ^b^	2.27 ^a^	0.62 ^a^	−27.30 ^a^	−26.96 ^a bc^
Baiyan 7	0.89 ^a^	8.37 ^bc^	125.58 ^b^	3.13 ^ab^	2.54 ^a^	0.57 ^a^	−26.63 ^b^	−26.65 ^c d^
Daoke	0.92 ^a^	9.79 ^d^	122.00 ^b^	3.38 ^b^	2.72 ^ab^	0.61 ^a^	−26.81 ^b^	−27.08 ^ab^
Jiayan 2	0.88 ^a^	7.93 ^ab^	129.33 ^b^	3.11 ^ab^	2.24 ^a^	0.60 ^a^	−26.35 ^b^	−26.53 ^d^
**Treatments(T)**								
T_0_ (Control)	1.40 ^b^	17.22 ^d^	253.3 ^c^	5.33 ^c^	3.37 ^b^	0.65 ^b^	−28.03 ^a^	−27.13 ^a^
T_1_ (8 dS m^−1^)	0.76 ^a^	8.68 ^c^	100.8 ^b^	3.16 ^b^	3.00 ^b^	0.61 ^b^	−26.95 ^b^	−26.72 ^bc^
T_2_ (16 dS m^−1^)	0.71 ^a^	5.39 ^b^	98.4 ^b^	2.82 ^b^	1.96 ^a^	0.56 ^a^	−26.08 ^c^	−26.63 ^c^
T_3_ (24 dS m^−1^)	0.66 ^a^	2.72 ^a^	60.8 ^a^	1.57 ^a^	1.82 ^a^	0.56 ^a^	−26.04 ^c^	−26.95 ^ab^
**Level of Significance**								
G	0.875	0.000	0.000	0.001	0.001	0.473	0.000	0.000
T	0.000	0.000	0.000	0.000	0.000	0.004	0.000	0.000
G × T	0.991	0.000	0.000	0.000	0.099	0.000	0.081	0.665

**Table 4:** Genotype and treatment effects on nitrogen isotope composition (‰) of shoots (δ^15^N_shoot_) and roots (δ^15^N_root_), nitrogen concentration of shoots (N_shoot_) and roots (N_root_), glutamine synthetase activity (GS activity), nitrate reductase activity (NR) and leaf chlorophyll content (Chl) of six oat genotypes in various salinity conditions. Genotype values are the means of 12 measurements (four treatments and three replicates per treatment), while treatment values are the means of the 18 measurements (six genotypes and three replicates per genotype). Means followed by different letters are significantly different (*p* < 0.05) according to Tukey’s-b test.

	δ^15^N_shoot_ (‰)	δ^15^N_root_ (‰)	N_shoot_ (%)	N_root_ (%)	Chl (SPAD Units)	NR Activity (μmol NO_2_^−^ g^−1^ FW h^−1^)	GS Activity (OD g^−1^ FW h^−1^)
**Genotypes (G)**							
Dahan	−3.03 ^a^	−4.42 ^a^	3.66 ^a^	2.04 ^ab^	43.15 ^a^	4.09 ^bc^	5.02 ^a^
Musile	−3.32 ^a^	−3.43 ^b^	3.84 ^ab^	2.00 ^ab^	50.60 ^b^	4.41 ^c^	5.11 ^a^
Tianyan 1	−2.80 ^a^	−4.49 ^a^	4.01 ^b^	1.94 ^ab^	44.83 ^a^	2.78 ^a^	5.09 ^a^
Baiyan 7	−3.01 ^a^	−3.74 ^ab^	3.74 ^a^	1.79 ^a^	48.16 ^b^	3.78 ^b^	4.94 ^a^
Daoke	−2.97 ^a^	−4.57 ^a^	3.77 ^a^	2.15 ^b^	49.08 ^b^	4.94 ^d^	4.42 ^a^
Jiayan 2	−3.14 ^a^	−3.61 ^ab^	3.73 ^a^	1.86 ^ab^	43.96 ^a^	4.32 ^ab^	4.55 ^a^
**Treatments (T)**							
T_0_ (Control)	−2.04 ^c^	−3.05 ^b^	4.62 ^d^	2.16 ^b^	48.18 ^b^	6.42 ^d^	6.16 ^d^
T_1_ (8 dS m^−1^)	−2.95 ^b^	−4.01 ^a^	3.80 ^c^	1.97 ^ab^	46.76 ^b^	4.80 ^c^	5.45 ^c^
T_2_ (16 dS m^−1^)	−3.63 ^a^	−4.54 ^a^	3.53 ^b^	1.79 ^a^	47.82 ^b^	3.76 ^b^	4.50 ^b^
T_3_ (24 dS m^−1^)	−3.59 ^a^	−4.59 ^a^	3.22 ^a^	1.93 ^a^	43.76 ^a^	1.22 ^a^	3.31 ^a^
**Level of Significance**							
G	0.544	0.002	0.001	0.024	0.000	0.000	0.027
T	0.000	0.000	0.000	0.001	0.000	0.000	0.000
G × T	0.244	0.169	0.460	0.563	0.002	0.000	0.006

**Table 5:** Genotype and treatment effects on ion concentration of shoots and roots of six oat genotypes grown under different combinations of salinity. Genotype values are the means of 12 measurements (four treatments and three replicates per treatment), while treatment values are the means of the 18 measurements (six genotypes and three replicates per genotype). Means followed by different letters are significantly different (*p* < 0.05) according to Tukey’s-b test. The units of Ca^2+^, Mg^2+^, K^+^, Na^+^ and *p* concentrations are mmol g^−1^ DM.

	Shoot							Root						
	Ca^2+^	Mg^2+^	K^+^	Na^+^	P	K^+^/Na^+^	Ca^2+^/Na^+^	Ca^2+^	Mg^2+^	K^+^	Na^+^	P	K^+^/Na^+^	Ca^2+^/Na^+^
**Genotypes (G)**														
Dahan	0.080 ^b^	0.074 ^ab^	1.253 ^a^	1.951 ^b^	0.145 ^a^	15.66 ^a^	0.924 ^a^	0.207 ^a^	0.071 ^a^	0.411 ^abc^	0.591 ^a^	0.148 ^a^	1.754 ^a^	0.93 ^a^
Musile	0.076 ^ab^	0.068 ^a^	1.246 ^a^	1.666 ^ab^	0.148 ^a^	24.53 ^a^	1.461 ^a^	0.223 ^a^	0.080 ^a^	0.486 ^bc^	0.721 ^a^	0.173 ^ab^	1.953 ^a^	1.02 ^a^
Tianyan 1	0.080 ^b^	0.080 ^b^	1.317 ^a^	1.668 ^ab^	0.171 ^b^	20.11 ^a^	1.152 ^a^	0.234 ^a^	0.076 ^a^	0.398 ^ab^	0.649 ^a^	0.184 ^b^	2.022 ^a^	1.44 ^ab^
Jiayan 2	0.063 ^a^	0.065 ^a^	1.274 ^a^	1.716 ^ab^	0.167 ^b^	41.50 ^b^	1.871 ^a^	0.254 ^a^	0.076 ^a^	0.378 ^a^	0.603 ^a^	0.171 ^ab^	2.072 ^a^	1.60 ^ab^
Daoke	0.072 ^ab^	0.068 ^a^	1.242 ^a^	1.492 ^a^	0.148 ^a^	16.20 ^a^	0.918 ^a^	0.213 ^a^	0.075 ^a^	0.496 ^c^	0.714 ^a^	0.163 ^ab^	1.916 ^a^	0.97 ^a^
Baiyan 7	0.065 ^a^	0.066 ^a^	1.252 ^a^	1.544 ^a^	0.159 ^ab^	22.28 ^a^	1.020 ^a^	0.263 ^a^	0.075 ^a^	0.379 ^a^	0.551 ^a^	0.160 ^ab^	2.243 ^a^	1.93 ^b^
**Treatments (T)**														
T_0_ (Control)	0.092 ^c^	0.088 ^b^	1.714 ^c^	0.023 ^a^	0.130 ^a^	91.564 ^b^	4.791 ^b^	0.452 ^c^	0.092^d^	0.692 ^c^	0.107 ^a^	0.155 ^a^	6.700 ^b^	4.632 ^b^
T_1_ (8 dS m^−1^)	0.061 ^a^	0.066 ^a^	1.262 ^b^	1.178 ^b^	0.156 ^b^	1.076 ^a^	0.052 ^a^	0.184 ^b^	0.080 ^c^	0.415 ^b^	0.743 ^b^	0.152 ^a^	0.561 ^a^	0.259 ^a^
T_2_ (16 dS m^−1^)	0.062 ^a^	0.062 ^a^	1.081 ^a^	1.925 ^c^	0.185 ^c^	0.599 ^a^	0.032 ^a^	0.134 ^a^	0.069 ^b^	0.298 ^a^	0.747 ^b^	0.169 ^a^	0.402 ^a^	0.186 ^a^
T_3_ (24 dS m^−1^)	0.076 ^b^	0.065 ^a^	1.000 ^a^	3.565^d^	0.154 ^b^	0.283 ^a^	0.021 ^a^	0.158 ^ab^	0.060 ^a^	0.295 ^a^	0.956 ^c^	0.190 ^b^	0.311 ^a^	0.177 ^a^
**Level of Significance**														
G	0.002	0.000	0.650	0.024	0.000	0.000	0.031	0.104	0.139	0.001	0.075	0.008	0.754	0.011
T	0.000	0.000	0.000	0.000	0.000	0.000	0.000	0.000	0.000	0.000	0.000	0.000	0.000	0.000
G × T	0.128	0.120	0.428	0.156	0.012	0.000	0.004	0.216	0.023	0.359	0.683	0.238	0.933	0.003

**Table 6:** Multiple linear regressions (stepwise) explaining biomass variation across genotypes and treatments based on water status-associated traits, N status-associated traits, the total traits, and the combination plus ion concentrations as independent variables. Levels of significant are as follows: * *p* < 0.05; ** *p* < 0.01 and *** *p* < 0.001.

Models	Final Stepwise	*R* ^2^
Water status traits ^†^	Biomass = 7.41 + 0.13 P_n_ + 0.17 *E* − 0.31 δ^13^C_Shoot_ + 0.47 δ^13^C_root_	0.82 *
N status traits ^‡^	Biomass = −1.38 + 0.81 N_shoot_ + 0.09 NR + 0.14 GS_activity_	0.79 *
Water and N traits combined ^§^	Biomass = 7.42 + 0.59 N_shoot_ + 0.54 P_n_ + 0.16 GS_activity_ + 0.31 δ^13^C_root_	0.83 **
Total parameters ^#^	Biomass = 9.91 − 0.42 Na^+^_shoot_ + 9.37 Mg^2+^_shoot_ − 0.47 Na^+^_root_ + 0.23 δ^13^C_root_ − 3.67 P_shoot_	0.90 ***

^†^ Traits related to water status included in the analysis are defined as in Table 2, including: P_n_, g_s_, *E*, C_i_/C_a_, δ^13^C_shoot_ and δ^13^C_root_. ^‡^ Traits related to nitrogen status are defined as in Table 3, including: δ^15^N_shoot_, δ^15^N_root_, N_shoot_, N_root_, Chl, GS and NR. ^§^ All the physiological traits mentioned above. ^#^ All the physiological traits mentioned above combined with the concentrations of ions, including Ca^2+^, Mg^2+^, K^+^ Na^+^ and P of shoots and roots.

## Supplementary Materials

The following are available online at https://www.mdpi.com/2223-7747/9/8/1025/s1, Table S1: Effect of different levels of salinity on the plant biomass, photosynthetic traits (P_n_, g_s_, E, WUE_i_), carbon isotope composition (δ^13^C_shoot_), leaf chlorophyll content (Chl), nitrate reductase activity (NR) and glutamine synthetase activity (GS) of six different forage-type oat (*Avena sativa* L.) genotypes. The data shown are the means of three replicates for each genotype in each treatment. Means followed by different letters are significantly different (*p* < 0.05). Table S2: Effect of different levels of salinity on the ion concentration (P_shoot_, K^+^/Na^+^_shoot_, Ca^2+^/Na^+^_shoot_, Mg^2+^_root_, Ca^2+^/Na^+^_root_) of shoots and roots of six different forage-type oat (*Avena sativa* L.) genotypes. The data shown are the means of three replicates for each genotype in each treatment. Means followed by different letters are significantly different (*p* < 0.05).

## References

[1] MARA (Ministry of Agriculture and Rural Affairs of P.R.C.), 2018. http://www.moa.gov.cn/xw/bmdt/201801/t20180129_6135949.htm.

[2] Yang C., Wang G., Wang M. (2017). Production and trade of wild oat forage in China. Pratacult. Sci..

[3] Chawade A., Lindén P., Bräutigam M., Jonsson R., Jonsson A., Moritz T., Olsson O. (2012). Development of a model system to identify differences in spring and winter oat. PLoS ONE.

[4] Nevo E., Chen G. (2010). Drought and salt tolerances in wild relatives for wheat and barley improvement. Plant Cell Environ..

[5] Munns R., James R.A., Läuchli A. (2006). Approaches to increasing the salt tolerance of wheat and other cereals. J. Exp. Bot..

[6] Chamekh Z., Ayadi S., Karmous C., Trifa Y., Amara H., Boudabbous K., Yousfi S., Serret M.D., Araus J.L. (2016). Comparative effect of salinity on growth, grain yield, water use efficiency, δ^13^C and δ^15^N of landraces and improved durum wheat varieties. Plant Sci..

[7] Yousfi S., Serret M.D., Márquez A.J., Voltas J., Araus J.L. (2012). Combined use of δ^13^C, δ^18^O and δ^15^N tracks nitrogen metabolism and genotypic adaptation of durum wheat to salinity and water deficit. New Phytol..

[8] Yousfi S., Serret M.D., Araus J.L. (2013). Comparative response of δ^13^C, δ^18^O and δ^15^N in durum wheat exposed to salinity at the vegetative and reproductive stages. Plant Cell Environ..

[9] Aranjuelo I., Cabrera-Bosquet L., Morcuende R., Avice J.C., Nogués S., Araus J.L., Martínez-Carrasco R., Pérez P. (2011). Does ear C sink strength contribute to overcoming photosynthetic acclimation of wheat plants exposed to elevated CO_2_?. J. Exp. Bot..

[10] Zhou B., Serret M.D., Elazab A., Bort Pie J., Araus J.L., Aranjuelo I., Sanz-Sáez Á. (2016). Wheat ear carbon assimilation and nitrogen remobilization contribute significantly to grain yield. J. Integr. Plant Biol..

[11] Carillo P., Mastrolonardo G., Nacca F., Parisi D., Verlotta A., Fuggi A. (2008). Nitrogen metabolism in durum wheat under salinity: Accumulation of proline and glycine betaine. Funct. Plant Biol..

[12] Botella M.A., Cruz C., Martins-Louçao M.A., Cerdá A. (1993). Nitrate reductase activity in wheat seedlings as affected by NO^3−^/NH^4+^ ratio and salinity. J. Plant Physiol..

[13] Irshad M., Honna T., Eneji A., Yamamoto S. (2002). Wheat response to nitrogen source under saline conditions. J. Plant Nutr..

[14] Debouba M., Maâroufi-Dghimi H., Suzuki A., Ghorbel M.H., Gouia H. (2007). Changes in growth and activity of enzymes involved in nitrate reduction and ammonium assimilation in tomato seedlings in response to NaCl stress. Ann. Bot..

[15] Sanz-Luque E., Chamizo-Ampudia A., Llamas A., Galvan A., Fernandez E. (2015). Understanding nitrate assimilation and its regulation in microalgae. Front. Plant Sci..

[16] Eisenberg D., Gill H.S., Pfluegl G.M., Rotstein S.H. (2000). Structure–function relationships of glutamine synthetases. Biochim. Biophys. Acta (BBA) Protein Struct. Mol. Enzymol..

[17] Cernusak L.A., Winter K., Turner B.L. (2009). Plant δ15N correlates with the transpiration efficiency of nitrogen acquisition in tropical trees. Plant Physiol..

[18] Craine J.M., Brookshire E., Cramer M.D., Hasselquist N.J., Koba K., Marin-Spiotta E., Wang L. (2015). Ecological interpretations of nitrogen isotope ratios of terrestrial plants and soils. Plant Soil.

[19] Serret M., Ortiz-Monasterio I., Pardo A., Araus J. (2008). The effects of urea fertilisation and genotype on yield, nitrogen use efficiency, δ15N and δ13C in wheat. Ann. Appl. Biol..

[20] Evans R., Bloom A., Sukrapanna S., Ehleringer J. (1996). Nitrogen isotope composition of tomato (*Lycopersicon esculentum* Mill. cv. T-5) grown under ammonium or nitrate nutrition. Plant Cell Environ..

[21] Liu T., Wang B., Xiao H., Wang R., Yang B., Cao Q., Cao Y. (2018). Differentially improved soil microenvironment and seedling growth of Amorpha fruticosa by plastic, sand and straw mulching in a saline wasteland in northwest China. Ecol. Eng..

[22] Baltruschat H., Fodor J., Harrach B.D., Niemczyk E., Barna B., Gullner G., Janeczko A., Kogel K.H., Schäfer P., Schwarczinger I. (2008). Salt tolerance of barley induced by the root endophyte *Piriformospora indica* is associated with a strong increase in antioxidants. New Phytol..

[23] Yousfi S., Serret M.D., Voltas J., Araus J.L. (2010). Effect of salinity and water stress during the reproductive stage on growth, ion concentrations, Δ^13^C, and δ^15^N of durum wheat and related amphiploids. J. Exp. Bot..

[24] Zhao G., Ma B., Ren C. (2007). Growth, gas exchange, chlorophyll fluorescence, and ion content of naked oat in response to salinity. Crop Sci..

[25] Ruan Y., Hu Y., Schmidhalter U. (2008). Insights on the role of tillering in salt tolerance of spring wheat from detillering. Environ. Exp. Bot..

[26] Wu H., Shabala L., Barry K., Zhou M., Shabala S. (2013). Ability of leaf mesophyll to retain potassium correlates with salinity tolerance in wheat and barley. Physiol. Plant..

[27] Zan W., Geng Z., Xue-min W., Hong-wen G. (2011). Growth, ion content and photosynthetic responses of two *Elytrigia* Desv. species seedlings to salinity stress. Afr. J. Biotechnol..

[28] Sun Z., Ren L., Fan J., Li Q., Wang K., Guo M., Wang L., Li J., Zhang G., Yang Z. (2016). Salt response of photosynthetic electron transport system in wheat cultivars with contrasting tolerance. Plant Soil Environ..

[29] Kiani-Pouya A., Rasouli F., Bazihizina N., Zhang H., Hedrich R., Shabala S. (2019). A large-scale screening of quinoa accessions reveals an important role of epidermal bladder cells and stomatal patterning in salinity tolerance. Environ. Exp. Bot..

[30] Barbour M.M., Warren C.R., Farquhar G.D., Forrester G., Brown H. (2010). Variability in mesophyll conductance between barley genotypes, and effects on transpiration efficiency and carbon isotope discrimination. Plant Cell Environ..

[31] Rajabi A., Ober E.S., Griffiths H. (2009). Genotypic variation for water use efficiency, carbon isotope discrimination, and potential surrogate measures in sugar beet. Field Crop. Res..

[32] Rebetzke G., Condon A.G., Richards R., Farquhar G. (2002). Selection for reduced carbon isotope discrimination increases aerial biomass and grain yield of rainfed bread wheat. Crop Sci..

[33] Morgan J.A., LeCain D.R., McCaig T.N., Quick J.S. (1993). Gas exchange, carbon isotope discrimination, and productivity in winter wheat. Crop Sci..

[34] Agastian P., Kingsley S., Vivekanandan M. (2000). Effect of salinity on photosynthesis and biochemical characteristics in mulberry genotypes. Photosynthetica.

[35] Elsheery N.I., Cao K.-F. (2008). Gas exchange, chlorophyll fluorescence, and osmotic adjustment in two mango cultivars under drought stress. Acta Physiol. Plant..

[36] Hirel B., Chardon F., Durand J. (2007). The contribution of molecular physiology to the improvement of nitrogen use efficiency in crops. J. Crop Sci. Biotechnol..

[37] Katiyar S., Dubey R. (1992). Influence of NaCl salinity on behaviours of nitrate reductase and nitrite reductase in rice seedlings differing in salt tolerance. J. Agron. Crop Sci..

[38] Reda M., Migocka M., Kłobus G. (2011). Effect of short-term salinity on the nitrate reductase activity in cucumber roots. Plant Sci..

[39] Botella M.A., Martínez V., Nieves M., Cerdá A. (1997). Effect of salinity on the growth and nitrogen uptake by wheat seedlings. J. Plant Nutr..

[40] Ullrich W.R. (2002). Salinity and nitrogen nutrition. Salinity: Environment-Plants-Molecules.

[41] Parry M.A., Reynolds M., Salvucci M.E., Raines C., Andralojc P.J., Zhu X.-G., Price G.D., Condon A.G., Furbank R.T. (2011). Raising yield potential of wheat. II. Increasing photosynthetic capacity and efficiency. J. Exp. Bot..

[42] Farquhar G.D., von Caemmerer S.V., Berry J. (1980). A biochemical model of photosynthetic CO_2_ assimilation in leaves of C_3_ species. Planta.

[43] Zhou B., Serret M.D., Pie J.B., Shah S.S., Li Z. (2018). Relative contribution of nitrogen absorption, remobilization, and partitioning to the ear during grain filling in chinese winter wheat. Front. Plant Sci..

[44] Yousfi S., Serret M.D., Araus J.L. (2009). Shoot δ15N gives a better indication than ion concentration or Δ13C of genotypic differences in the response of durum wheat to salinity. Funct. Plant Biol..

[45] Robinson D. (2001). δ15N as an integrator of the nitrogen cycle. Trends Ecol. Evol..

[46] Zhou B., Sanz-Sáez Á., Elazab A., Shen T., Sánchez-Bragado R., Bort J., Serret M.D., Araus J.L. (2014). Physiological traits contributed to the recent increase in yield potential of winter wheat from Henan Province, China. J. Integr. Plant Biol..

[47] Liu C. (2018). Study on Agronomic Traits and Cropping Patterns of Forage Oats in Western Jilin Province.

[48] Hoagland D.R., Arnon D.I. (1950). The water-culture method for growing plants without soil. Circ. Calif. Agric. Exp. Stn..

[49] Al-Tamimi N., Brien C., Oakey H., Berger B., Saade S., Ho Y.S., Schmöckel S.M., Tester M., Negrão S. (2016). Salinity tolerance loci revealed in rice using high-throughput non-invasive phenotyping. Nat. Commun..

[50] Condon A.G., Richards R., Rebetzke G., Farquhar G. (2002). Improving intrinsic water-use efficiency and crop yield. Crop Sci..

[51] Farquhar G.D., Ehleringer J.R., Hubick K.T. (1989). Carbon isotope discrimination and photosynthesis. Annu. Rev. Plant Biol..

