# Supplementary material for: Comparative Study of the Effects of Salinity on Growth, Gas Exchange, N Accumulation and Stable Isotope Signatures of Forage Oat (Avena sativa L.) Genotypes
Source: Plants (Basel). 2020 Aug 13;9(8):1025. doi: 10.3390/plants9081025 (PMC7464733; doi:10.3390/plants9081025)
Supplement: Supplementary file 1 [file plants-09-01025-s001.pdf]

Table S1. Effect of different levels of salinity on the plant biomass, photosynthetic traits (P_n, g_s, E, WUE_i), carbon isotope composition ($\delta^{13}C_{shoot}$), leaf chlorophyll content (Chl), nitrate reductase activity (NR) and glutamine synthetase activity (GS) of six different forage-type oat (*Avena sativa* L.) genotypes. The data shown are the means of three replicates for each genotype in each treatment. Means followed by different letters are significantly different ($P < 0.05$).

	**Treatments**	**Dahan**	**Musile**	**Tianyan 1**	**Baiyan 7**	**Daoke**	**Jiayan 2**	**Genotype (*P*-value)**
Plant biomass (g plant^{-1})	T0	3.46a	3.63ab	3.73ab	3.93bc	4.22cd	4.38d	0.000
	T1	2.84	2.78	2.98	3.08	2.86	2.9	0.149
	T2	2.14a	2.28ab	2.79b	2.4ab	2.46ab	2.11a	0.023
	T3	1.72	1.54	1.64	2.0	1.69	1.81	0.154
P_n (mol m^{-2} s^{-1})	T0	16.53b	14.23a	21.30c	16.47b	17.10b	17.73b	0.000
	T1	5.23a	12.43c	4.50a	5.83a	15.63d	8.50d	0.000
	T2	5.60b	4.40a	6.40b	8.43a	3.70a	3.83a	0.000
	T3	3.30b	3.00b	2.93b	2.73b	2.73b	1.67a	0.001
Gs (mmol m^{-2} s^{-1})	T0	206.67a	201.67a	370.33d	260.33b	188.67a	292.33c	0.000
	T1	39.33a	191.33d	77.33b	68.33b	142.33c	86.33b	0.000
	T2	67.00a	102.33b	126.67c	105b	95.00b	94.67b	0.000
	T3	49.00a	81.00c	60.33b	68.67b	62.00b	44.00a	0.000
E (mmol m^{-2} s^{-1})	T0	4.53a	4.70a	7.63b	5.37a	4.00a	5.77ab	0.006
	T1	1.23a	5.37b	1.87a	1.97a	5.50b	3.03a	0.000
	T2	2.27	2.6	3.53	3.53	2.57	2.47	0.117
	T3	1.67	2	1.5	1.67	1.43	1.17	0.241
WUE_i	T0	3.92 ab	3.09 ab	2.82 b	3.09 ab	4.28 a	3.08 ab	0.022
	T1	4.30 a	2.49 b	2.48 b	2.99 ab	2.95 ab	2.81 ab	0.022
	T2	2.49	2.00	1.84	2.44	1.44	1.57	0.188
	T3	2.05	1.51	1.96	1.67	2.23	1.50	0.430
$\delta^{13}C_{shoot}$ (‰)	T0	-27.88	-28.5	-28.4	-28.21	-27.69	-27.54	0.118
	T1	-26.56	-27.25	-27.36	-26.75	-27.1	-26.73	0.233
	T2	-25.67b	-25.91b	-26.79a	-25.98b	-26.42ab	-25.75b	0.006
	T3	-26.78	-25.7	-26.67	-25.62	-26.05	-25.42	0.028
Chl (SPAD readings)	T0	44.50a	54.30c	44.77a	47.60ab	52.33bc	45.63a	0.001
	T1	44.33	48.97	45.43	50.03	47.93	43.87	0.098

	T2	43.50a	48.47ab	49.53b	47.37ab	50.90b	47.20ab	0.011
	T3	40.30a	50.67c	39.60a	47.67bc	45.17b	39.17a	0.000
NR activity (μmol g^{-1} h^{-1})	T0	7.10bc	5.63ab	4.77a	7.11bc	8.10c	5.82ab	0.000
	T1	4.16b	5.89c	2.97a	4.99bc	5.75c	5.08ab	0.000
	T2	3.55bc	4.76cd	1.83a	2.64ab	4.59cd	5.21d	0.000
	T3	1.55b	1.38b	1.54b	0.38a	1.32b	1.19b	0.003
GS activity (OD g^{-1} h^{-1})	T0	7.25b	5.85a	5.61a	6.79ab	5.81a	5.65a	0.014
	T1	5.17ab	6.41b	6.26b	5.21ab	4.31ab	5.33ab	0.011
	T2	4.79	4.81	4.57	4.38	4.09	4.36	0.778
	T3	2.87	3.37	3.91	3.37	3.48	2.87	0.294

Table S2. Effect of different levels of salinity on the ion concentration (P_{shoot}, K^+/Na^+_{shoot}, Ca^{2+}/Na^+_{shoot}, Mg^{2+}_{root}, Ca^{2+}/Na^+_{root}) of shoots and roots of six different forage-type oat (*Avena sativa* L.) genotypes. The data shown are the means of three replicates for each genotype in each treatment. Means followed by different letters are significantly different ($P < 0.05$).

	Treatments	Dahan	Musile	Tianyan 1	Baiyan 7	Daoke	Jiayan 2	Genotype (*P*-value)
P_{shoot} (mmol g^{-1})	T0	0.13bc	0.12ab	0.14bc	0.13bc	0.11a	0.15c	0.003
	T1	0.15	0.16	0.15	0.15	0.16	0.17	0.599
	T2	0.16a	0.16a	0.22b	0.20ab	0.18ab	0.19ab	0.011
	T3	0.14a	0.15ab	0.17b	0.16ab	0.15ab	0.16ab	0.092
K^+/Na^+_{shoot}	T0	60.95a	96.24a	78.37a	87.08a	62.71a	164.03b	0.006
	T1	1.01	1.07	1.08	1.04	1.18	1.09	0.450
	T2	0.40a	0.54ab	0.72b	0.71b	0.61ab	0.62ab	0.027
	T3	0.29	0.28	0.27	0.29	0.3	0.27	0.947
Ca^{2+}/Na^+_{shoot}	T0	3.59	5.74	4.49	3.98	3.56	7.39	0.072
	T1	0.05ab	0.05a	0.06b	0.05a	0.06ab	0.04a	0.004
	T2	0.03ab	0.03ab	0.04b	0.03ab	0.03a	0.03ab	0.061
	T3	0.02	0.02	0.02	0.02	0.02	0.02	0.139
Mg^{2+}_{root} (mmol/g)	T0	0.09	0.1	0.1	0.09	0.09	0.08	0.402
	T1	0.06a	0.08b	0.08b	0.09b	0.08b	0.09b	0.003
	T2	0.07	0.07	0.07	0.07	0.06	0.07	0.488
	T3	0.06	0.07	0.06	0.05	0.06	0.06	0.225
Ca^{2+}/Na^+_{root}	T0	3.11	3.51	5.21	6.97	3.39	5.59	0.055
	T1	0.2	0.23	0.2	0.38	0.17	0.37	0.022
	T2	0.19	0.18	0.18	0.17	0.18	0.22	0.834
	T3	0.22	0.15	0.17	0.19	0.12	0.22	0.229
